# Mechanisms in AIT: Insights 2021 

**DOI:** 10.5414/ALX02300E

**Published:** 2022-11-21

**Authors:** Pattraporn Satitsuksanoa, Alba Angelina, Oscar Palomares, Mübeccel Akdis

**Affiliations:** 1 Swiss Institute of Allergy and Asthma Research (SIAF), University of Zurich, Davos, Switzerland, and; 2 Department of Biochemistry and Molecular Biology, School of Chemistry, Complutense University of Madrid, Spain; * The authors contribute equally as first authors.; ** The authors contribute equally as last authors.

**Keywords:** allergen-specific immunotherapy (AIT), regulatory T cells (Tregs), regulatory B cells (Bregs), regulatory innate lymphoid cells (ILCregs), allergen-specific antibodies

## Abstract

Background: Allergen-specific immunotherapy (AIT) is currently the only treatment with potential long-term disease-modifying effects for patients suffering from allergic diseases such as allergic rhinitis, allergic asthma, venom allergy, or IgE-mediated food allergy. A better understanding of the molecular mechanisms underlying immune responses during successful AIT is of utmost importance and it may help to develop more effective and safer treatments. Materials and methods: PubMed literature review was performed using keywords such as allergen-specific immunotherapy; regulatory T cells; regulatory B cells; regulatory innate lymphoid cells; and allergen-specific antibody from years 2018 to 2021. Results: The proposed mechanism of long-term tolerance induction in AIT, even upon treatment discontinuation, involves basophils, mast cells, innate lymphoid cells, dendritic cells, allergen-specific regulatory T and B cells, downregulation of effector type 2 responses, decrease in the production of IgE and increase in production of allergen-specific blocking antibodies, such as IgG2 and IgG4. Conclusion: We summarize the most recent advances related to mechanisms involved in the restoration of healthy immune responses to allergens during AIT. Our knowledge in this regard has significantly improved over the last years, which might well contribute to design novel and improved therapeutic approaches.

## Introduction 

Allergen-specific immunotherapy (AIT) is currently the only treatment that might influence the natural course of allergic diseases such as IgE-mediated food allergy, venom allergy, allergic rhinitis, or allergic asthma [1]. During the past decades, the prevalence of allergic diseases has dramatically increased worldwide, thus becoming one of the top global health issues. During this time, we have witnessed significant advances in the knowledge of the immunological mechanisms underlying allergic immune responses as well as those involved in allergen-specific tolerance induction. In this manuscript, we summarize the basic mechanisms underlying allergic diseases and highlight the immune mechanisms of action together with the up-to-date advancements in AIT. 

## Cellular and molecular mechanisms underlying successful AIT 

Allergic inflammation is initiated when allergens are captured by antigen-presenting cells (APCs) in the skin, respiratory or gastrointestinal mucosa. Epithelial cells produce alarmins such as IL-25, IL-33, and TSLP in response to allergens and other environmental insults. These allergens are subsequently processed into peptides and presented by APCs to naïve CD4^+^ T cells, which differentiate into T helper (Th) 2 cells and release type 2 cytokines such as IL-4, IL-5, IL-9, and IL-13. B cells are triggered to undergo IgE class-switching and differentiate into plasma cells secreting large amounts of allergen-specific IgE antibodies that bind to the high-affinity IgE receptor (FcεRI) on the surface of mast cells and basophils. Allergen re-exposure induces IgE-FcεRI complexes cross-linking, leading to the release of anaphylactogenic mediators such as histamine, leukotrienes, and cytokines. These mediators contribute to type-1 hypersensitivity reactions and occasionally lead to anaphylactic reactions. Late-phase reactions and multiple allergen exposures perpetuate allergic inflammation and contribute to tissue remodeling, fibrosis, and the most chronic manifestations of allergic diseases [1, 2]. 

To better understand the allergic immune cascade and tolerance induction mechanisms, the role of several immune cells including mast cells, basophils, innate lymphoid cells (ILCs), dendritic cells (DCs), regulatory T cells (Tregs), and regulatory B cells (Bregs) needs to be extensively monitored and investigated during the course of AIT and after treatment discontinuation. 

## Mast cells and basophils 

Mast cells and basophils play a key role in the effector phases of allergy. The allergen-dependent cross-linking of the IgE bound to FcεRI on mast cells and basophils triggers the release of different mediators and type 2 cytokines that promote allergic inflammation. AIT induces very rapid desensitization of mast cells and basophils evidenced by low responsiveness to allergens despite the high allergen-specific IgE levels observed at the initiation of the treatment. Late effects of AIT are associated with a reduction of mast cell and basophil infiltration in the tissues and a reduced mediator release [3]. One possible mechanism is the increased production of allergen-specific IgG4 and the elevated expression of the low-affinity IgG receptor (FcγRIIa and FcγRIIb) on mast cells and basophils. IgG4 competes with IgE in allergen binding, but also the binding of the FcγRIIb receptor by allergen-IgG complexes suppresses the IgE-mediated activation on mast cells. IgG-mediated inhibition also reduces Th2 cytokine release from mast cells and basophils. [4] 

Another mechanism is the quick upregulation of histamine receptor 2, which has an inhibitory effect on FcεRI-mediated activation and degranulation of basophils. The decrease of basophil responsiveness has been documented during several studies of immunotherapy [5]. Interestingly, the analysis of basophil responses before, during, and after AIT can help to identify transient desensitization or maintained unresponsiveness. 

## Innate lymphoid cells 

ILCs are recently described cells of the innate immune system. They are classified into two main prominent subsets that are cytotoxic and non-cytotoxic (helper) ILCs. The cytotoxic ILCs consist of natural killer (NK) cells that display functions of CD8^+^ cytotoxic T cells and lymphoid tissue inducer (LTi) cells that are involved in secondary lymphoid structure development. The helper ILCs have been subsequently separated into three different phenotypes: group 1 ILCs (ILC1s), group 2 ILCs (ILC2s), and group 3 ILCs (ILC3s). These three different subsets resemble the functions of Th1, Th2, and Th17 cells, respectively. Interestingly, new discoveries of IL-10-producing ILCs and retinoic acid (RA]-mediated pathway of IL-10-producing ILC2 induction has revealed the hidden immune regulatory properties of these cells [6, 7]. Golebski et al. [8] successfully demonstrated the insightful role of IL-10^+^ KLRG1^+^ ILC2s during AIT in patients with grass pollen allergy. Their results revealed that the competence of ILC2 to produce IL-10 was restored in patients who received grass-pollen sublingual immunotherapy. For this reason, IL-10^+^ ILC2s exhibit disease-modulating effects by AIT. 

## Dendritic cells 

**Figure 1:**
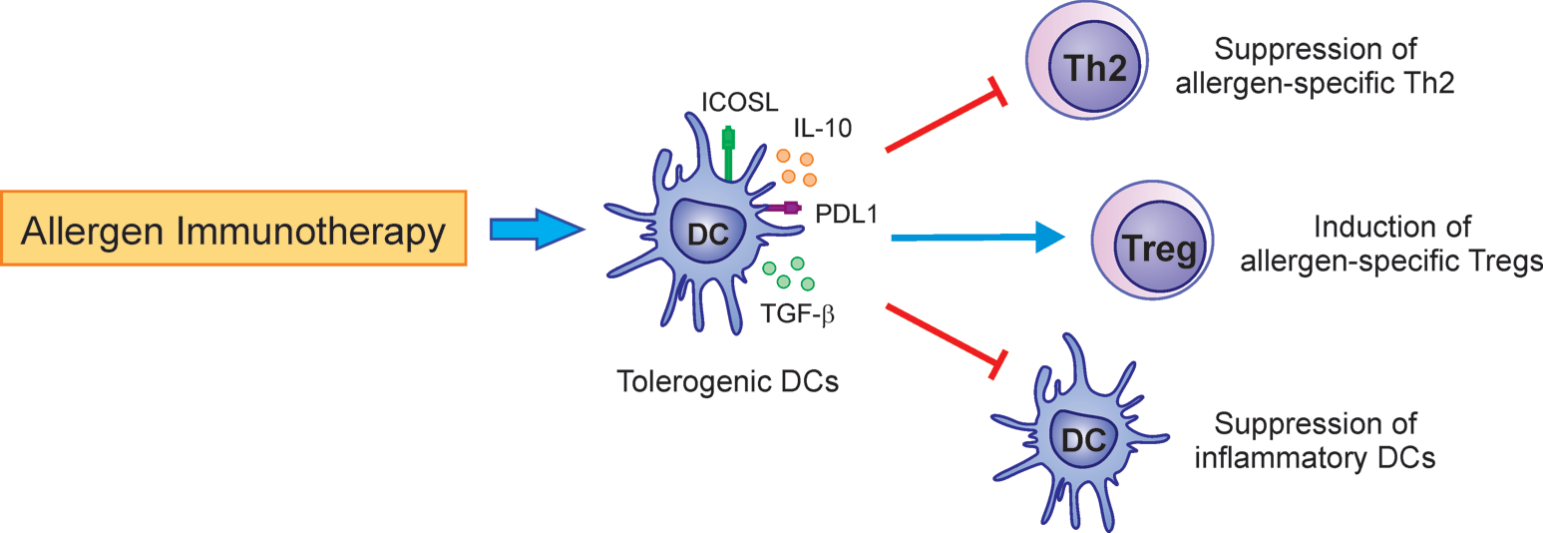
Mechanisms of immune tolerance induced by DCs during AIT. Tolerogenic DCs produce IL-10 and TGF-β, and express surface molecules that suppress Th2 and Th17 cells as well as inflammatory DCs, and induce allergen-specific Tregs.

DCs are APCs with the dual capacity to initiate and maintain allergic inflammation or to induce tolerance. DCs play an important role during the course of AIT. AIT induces an increase in plasmacytoid DCs (pDCs), which is a DC subset involved in the induction of Tregs and oral tolerance [9, 10]. A reduction in the frequency of CD1c^+^ conventional DCs (cDCs), which support Th2 responses in allergic patients, was also observed after AIT [9]. Mouse models have demonstrated that oral CD11b^+^ cDCs and macrophages transport sublingual allergens to lymph nodes and induce allergen-specific Tregs [3, 11]. Tolerogenic DCs represents a heterogeneous subset of DCs with an immature or mature phenotype that display increased capacity to produce IL-10. Allergoid-mannan conjugates generate tolerogenic IL-10-producing DCs and reprogram monocytes and macrophages into tolerogenic phenotypes [12, 13, 14]. Similarly, a nanovaccine produced by coupling PGLA-encapsulated ovalbumin with mannan induced in vitro IL-10-producing DCs that generated Tregs [15]. Cannabinoids also induce tolerogenic DCs able to generate Tregs by mechanisms depending on autophagy and metabolic reprogramming [16]. Another in vitro study showed the capacity of IL-27 produced by DCs to suppress grass pollen-stimulated peripheral blood mononuclear cell (PBMC) proliferation. Interestingly, a mouse model of allergic airway inflammation highlighted the critical role of IL-10 signaling in DCs to induce allergen-specific tolerance [17]. The scheme of tolerance-induced mechanisms by DCs during AIT is shown in Figure 1. 

## Regulatory T cells 

**Figure 2:**
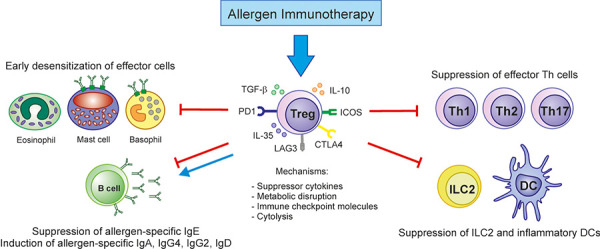
Mechanisms of immune tolerance induced by Tregs during AIT. Tregs produce immunosuppressive cytokines such as IL-10, TGF-β, and IL-35 that result in suppression of early desensitization of effector cells (eosinophils, mast cells, and basophils), effector Th cells (Th1, Th2, and Th17 cells), inflammatory DCs, ILC2, and allergen-specific IgE. Through IL-10, TGF-β, and IL-35 cytokine production, allergen-specific IgA, IgG2, IgG4, and IgD antibodies are released and exhibit IgE-blocking effects.

Induction and maintenance of peripheral tolerance to allergens require the fine balance between Tregs and effector cells. Peripheral T-cell tolerance is characterized by an increase of AIT-induced Tregs and the shift of Th2 cell responses to Th1 [3]. Allergen-specific Tregs can be divided into thymic or natural Tregs (tTregs) and inducible Tregs (pTregs) including FOXP3-expressing iTregs, IL-10-secreting Tr1 cells, and TGFβ-producing Th3 cells [18, 19]. The suppression of different cell subsets by Tregs is required to completely establish cell-mediated tolerance. Tregs generated during and after AIT suppress effector cell function and induce blocking antibodies [1]. In AIT, four different mechanisms of Treg suppression have been described: secretion of inhibitory cytokines such as IL-10, TGF-β, and IL-35; metabolic disruption mechanisms; mechanisms involving surface molecules including programmed death 1 (PD1), cytotoxic T-lymphocyte antigen 4 (CTLA-4), lymphocyte-activation gene 3 (LAG3), or inducible costimulatory molecule (ICOS); and cytolysis (Figure 2). Allergen-specific Tregs increase and Th2 cell reduction with the induction of natural or clinical tolerance in beekeepers during allergen exposure in the beekeeping season and AIT-treated allergic patients, respectively. Ex vivo analysis of human peripheral blood cells showed that house dust mite (HDM) AIT involves increase of functional allergen-specific Tregs and decrease of allergen-specific ILT3^+^ Treg cells, which display impaired suppressive function [20]. AIT generates long-term Treg production by inducing epigenetic changes in FOXP3 regions in Tregs. IL-10-producing Tregs were induced after birch and grass pollen AIT. Interestingly, IL-35-inducible Tregs have been identified as a subset of iTregs with the capacity to reduce Th2 inflammation, T cell proliferation, and cytokines produced by ILC2s. Recent studies have demonstrated that successful subcutaneous immunotherapy (SCIT) increases IL-35 and IL-35-induced Tregs in blood [3]. All these studies have demonstrated the critical role of the tolerance induced by allergen-specific Tregs in successful AIT in humans. 

## Regulatory B cells 

**Figure 3:**
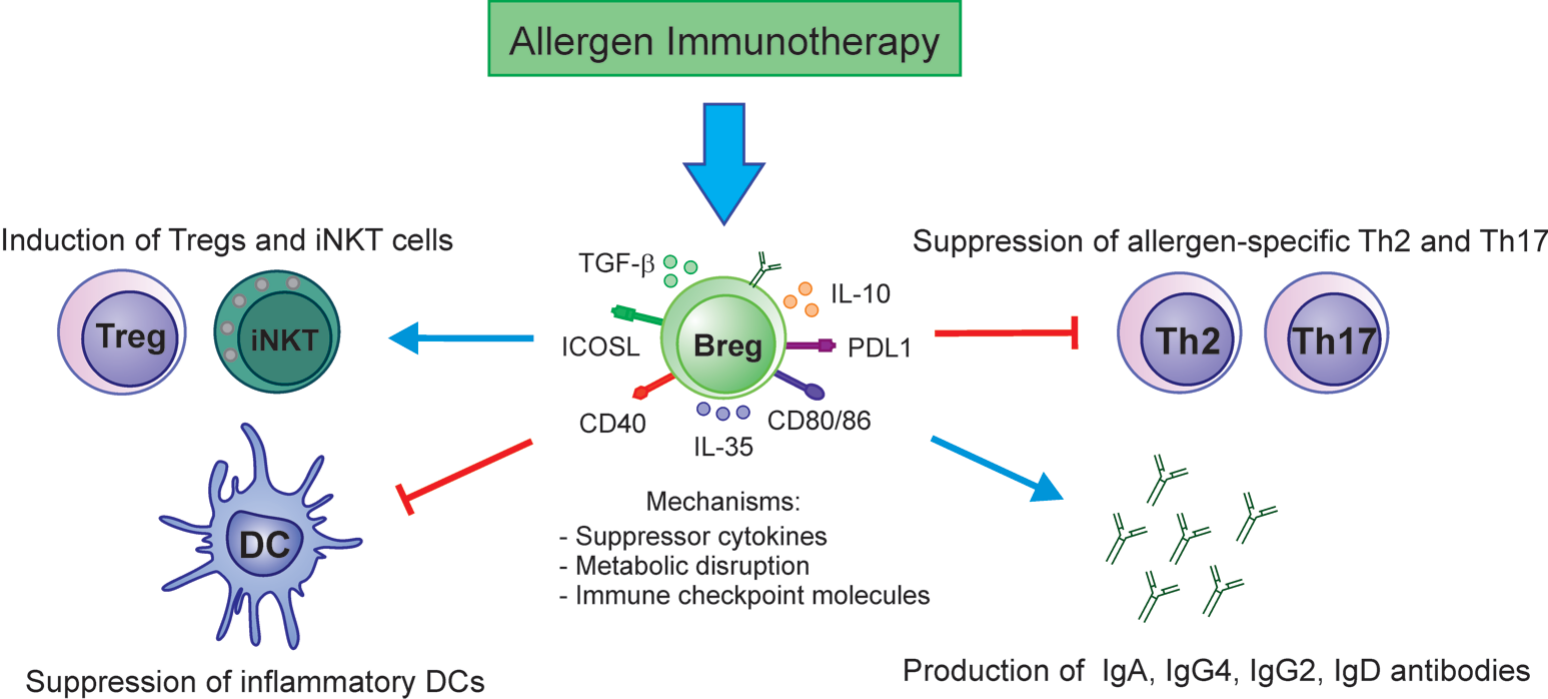
Mechanisms of immune tolerance induced by Bregs during AIT. Bregs suppress effector T cells (Th2 and Th17) and induce expansion of Treg cells via the release of IL-10, TGF-β, and IL-35 cytokines. Diverse surface molecules on Breg cells including BCR, PDL1, CD39, CD73, CD80/CD86, CD40, ICOS-L, and AhR are well-expressed and suppress the inflammatory responses. In autoimmune tolerance, Bregs activate iNKT cells with suppressive function. Moreover, Bregs are also the main producer of allergen-specific IgA, IgG2, IgG4, and IgD antibodies that compete with the crosslinking of allergen-specific IgE to effector cells.

Although B cells play a major role in the immune system via the production of specific antibodies, recent evidence clearly revealed that B cells can also mediate immune responses through alternative mechanisms beyond antibody production [21]. Bregs play a crucial role in secreting the immunosuppressive cytokines including IL-10, IL-35, and TGF-β, and expressing immunosuppressive receptors such as cell membrane-bound molecules like B cell receptor (BCR), PDL1, CD39, CD73, CD80/CD86, CD40, inducible costimulatory ligand (ICOSL), and aryl-hydrocarbon receptor (AhR) (Figure 3) [22]. Bregs can be stimulated by different factors, including inflammatory cytokines such as IL-6, IL-1β, and IFN-α, microbial compounds such as TLR4 or TLR9 ligands, along with CD40 ligation [23]. During AIT, IL-10-secreting B regulatory 1 (Br1) cells specific to bee venom allergen phospholipase A_2_ (PLA) suppress antigen-specific CD4^+^ T-cell proliferation and also produce IgG4 [24]. In the following study, IL-10^+^Br1 cells increased their frequency in allergic patients receiving AIT and naturally exposed to allergen [25]. In addition, immunotherapy-treated patients with HDM allergy exhibit a significantly increased population of Der p 1-specific B cells, plasmablasts, IL-10^+^, and IL-1RA^+^ Bregs [26]. Furthermore, Bregs present lipid antigen to sustain invariant natural Killer T cells (iNKT) cell homeostasis and activation in healthy individuals compared to systemic lupus erythematous (SLE) patients [27]. Indeed, Bregs play an essential role in achieving immune tolerance during AIT. 

## Allergen-specific IgE, IgG4, IgA, and IgD responses 

The secretion of immunoregulatory cytokine IL-10 from Tregs, Bregs, regulatory innate lymphoid cells (ILCregs) could suppress Th2 responses and shift towards the induction of allergen-specific IgA and IgG4 antibodies with inhibitory activity [28, 29, 30]. IgG4 antibodies attenuate allergic responses by inhibiting the activity of IgE to mast cell and basophil activation [31]. Similar to IgG4, an IgE-blocking effect has been observed in IgG2. Recent data reveals that both IgG2 and IgG4 are capable of suppressing IgE-mediated allergic responses in patients who received sublingual immunotherapy against grass pollen [32]. Although IgG2 and IgG4 are found to be mediating this protective effect in AIT [33], the tolerance induction from other IgG subclasses remains unclear. In order to clarify these roles of other IgG subclasses during AIT, further studies on this topic are required. Lastly, allergen-specific IgD was increased during the course of AIT in HDM-sensitized asthmatic patients [34]. In addition, allergen-specific IgD was increased in children consuming cow’s milk compared to children with cow’s milk elimination, suggesting that food antigens may establish humoral IgD responses in humans [35]. 

## Novel adjuvants 

**Figure 4:**
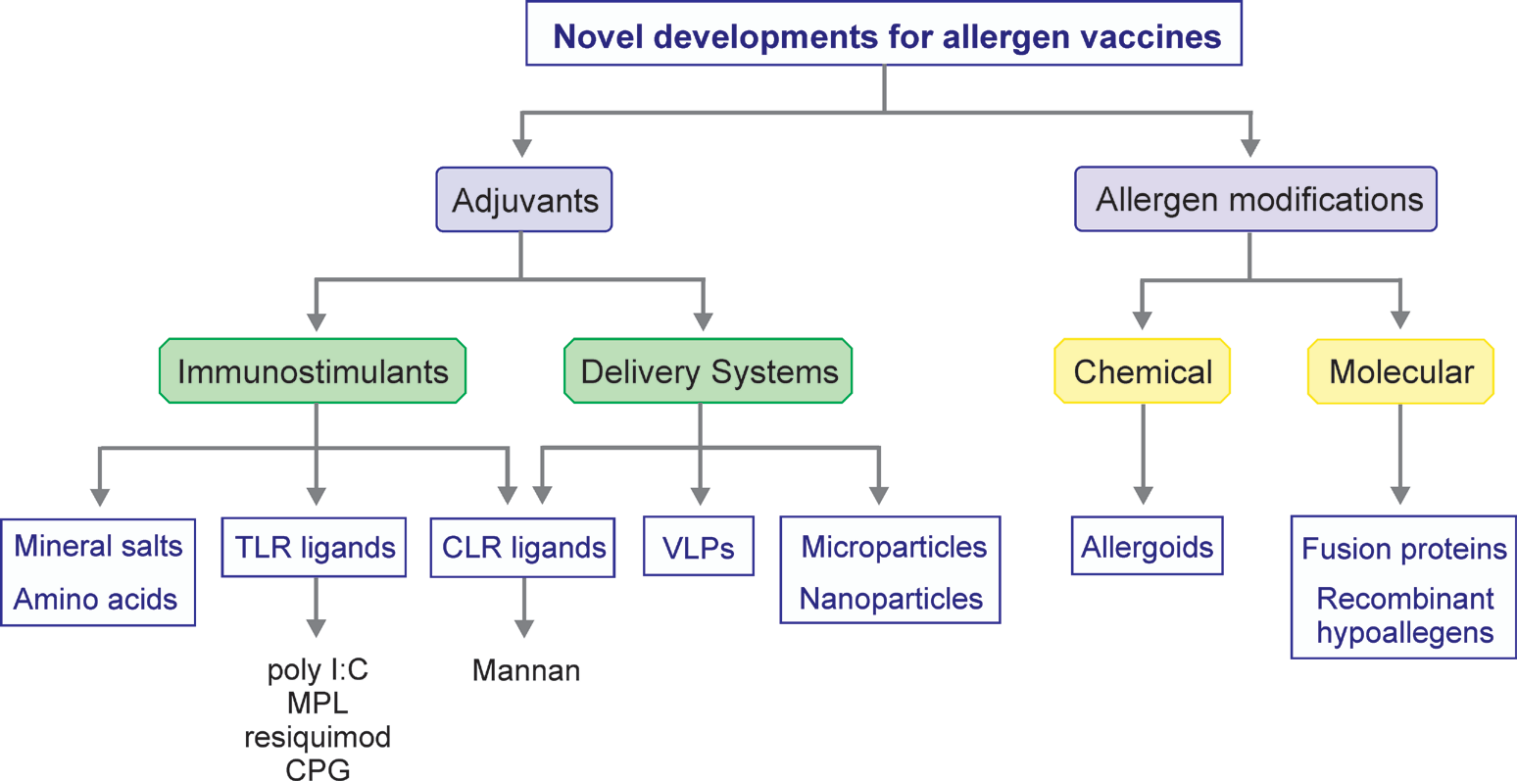
Novel adjuvants for AIT treatments. Adjuvants can be classified as immunostimulant substances including mineral salts, amino acids, TLR ligands or CLR ligands, or delivery systems such as VLPs or micro- or nanoparticles. Chemical and molecular modifications of allergens have also been developed for AIT.

AIT adjuvant must be safe, stable, and induce Th1/Treg cell responses. Aluminium hydroxide is the most widely used adjuvant in humans. It stimulates innate immune cells and creates a reservoir of antigen at the site of injection (depot effect). In recent years, more and more studies focus on developing novel adjuvants that can increase proper immune responses in target cells (immunomodulators) or increase the capture of allergens by APCs (delivery systems). Calcium phosphate, a mineral salt, and microcrystalline tyrosine, a biodegradable amino acid, are immunomodulators also used in current AIT products due to their depot effect [36]. Among immunomodulators, TLR ligands have been also used in allergy vaccination. For example, poly I:C (TLR3 ligand), monophosphoryl lipid A (TLR4 ligand), resiquimod (TLR7/8 ligand), or unmethylated CpG motifs (TLR9 ligand) have been proposed as adjuvants for AIT due to their capacity of promoting tolerance in different allergy models. In addition, the incorporation of flagellin (TLR5 ligand) into allergens is a new interesting approach. Novel approaches targeting C-type lectin receptors have been developed. Allergoid-mannan conjugates were demonstrated to enhance allergen uptake capacity and to induce strong regulatory responses, thus acting simultaneously as immunomodulatory and delivery system adjuvant (Figure 4) [12, 13, 14, 37]. Remarkably, aluminium hydroxide impairs the tolerogenic properties imprinted by allergoid-mannan conjugates in human DCs [38]. Among delivery systems, viral-like particles (VLPs), micro-particles, and nano-particles have been investigated because the encapsulation of allergens in these systems prevents allergen degradation, achieves high concentrations of allergens, and impairs IgE recognition. Currently, other strategies to obtain hypoallergenic derivates are widely investigated in clinical practice [36]. 

## Future perspectives of AIT vaccines 

Future perspectives of AIT vaccines include the development of new adjuvants, hypoallergenic allergoids, and recombinant allergens, new application routes, and a better understanding of the mechanisms underlying successful AIT. Recently, the concepts of “innate trained immunity” and trained immunity-based vaccines (TIbVs) were defined [39]. This describes the long-term functional reprogramming of innate immune cells which is induced by an insult and which promotes an altered response against a second insult. Mechanistically, trained immunity implies metabolic reprogramming and epigenetic changes. Trained immunity is clinically relevant when an exacerbated immune response drives disease progression, such as allergy. Therefore, targeting trained immunity could be a relevant therapeutic strategy for allergic diseases [40]. Recently, it has been demonstrated that allergoid-mannan conjugates can reprogram monocytes from both non-atopic and allergic donors into tolerogenic DCs via epigenetic and metabolic reprogramming [14]. Another recent study showed that SCIT for aeroallergens induces changes associated with tolerance in innate immune cells from allergic patients [9]. These data suggest a potential role of trained immunity in the mechanisms of tolerance induction during AIT. 

## Conclusion 

Successful AIT establishes the induction of immune tolerance toward allergens, diminishes allergic symptoms, and improves clinical treatments in patients. After AIT, local infiltration of basophils and mast cells is reduced. In addition, induction of tolerogenic DC phenotype promotes the increase of Tregs which skew the immune response from Th2 to Th1 and induce the rise of Bregs. Consequently, Treg-derived IL-10 and Bregs prompt B-cell isotype switch toward IgG4 production. The greater understanding of underpinning mechanisms of AIT has improved medical treatment efficiency and better clinical outcomes in allergic patients and might well help to design better therapies in the near future. 

## Funding 

M.A. has received research support from the Swiss National Science Foundation No. 310030-201053/320030-159870 and the Sean N Parker Center for Allergy and Asthma Research at Stanford University. O.P.’s lab is supported by grants SAF-2017-84978-R from MINECO (Spain) and PID2020-114396RB-I00 from Ministerio de Ciencia e Innovación (Spain). 

## Conflict of interest 

M.A. has received research grants from Swiss National Science Foundation, Bern; research grant from the Stanford University; Leading House for the Latin American Region, Seed Money Grant. She is in the Scientific Advisory Board member of Stanford University-Sean Parker Asthma Allergy Center, CA; Advisory Board member of LEO Foundation Skin Immunology Research Center, Kopenhagen; and Scientific Co-Chair of World Allergy Congress (WAC) Istanbul, 2022. O.P. received research grants from MINECO, Ministerio de Ciencia e Innovación, CAM, Inmunotek, and Novartis and fees for giving scientific lectures or participation in Advisory Boards from: Allergy Therapeutics, Amgen, AstraZeneca, Diater, Pfizer, GlaxoSmithKline, Inmunotek, Novartis, Sanofi-Genzyme, and Stallergenes. P.S. and A.A. declare no conflict of interest. 

## References

[1] van de Veen W Akdis M Tolerance mechanisms of allergen immunotherapy. Allergy. 2020; 75: 1017–1018. 31758812 10.1111/all.14126

[2] Głobińska A Boonpiyathad T Satitsuksanoa P Kleuskens M van de Veen W Sokolowska M Akdis M Mechanisms of allergen-specific immunotherapy: Diverse mechanisms of immune tolerance to allergens. Ann Allergy Asthma Immunol. 2018; 121: 306–312. 29966703 10.1016/j.anai.2018.06.026

[3] Celebi Sözener Z Mungan D Cevhertas L Ogulur I Akdis M Akdis C Tolerance mechanisms in allergen immunotherapy. Curr Opin Allergy Clin Immunol. 2020; 20: 591–601. 33002895 10.1097/ACI.0000000000000693

[4] Kanagaratham C El Ansari YS Lewis OL Oettgen HC IgE and IgG Antibodies as Regulators of Mast Cell and Basophil Functions in Food Allergy. Front Immunol. 2020; 11:603050. 33362785 10.3389/fimmu.2020.603050 PMC7759531

[5] Schmid JM Würtzen PA Siddhuraj P Jogdand P Petersen CG Dahl R Erjefält JS Hoffmann HJ Basophil sensitivity reflects long-term clinical outcome of subcutaneous immunotherapy in grass pollen-allergic patients. Allergy. 2021; 76: 1528–1538. 32145088 10.1111/all.14264

[6] Wang S Xia P Chen Y Qu Y Xiong Z Ye B Du Y Tian Y Yin Z Xu Z Fan Z Regulatory Innate Lymphoid Cells Control Innate Intestinal Inflammation. Cell. 2017; 171: 201–216.e18. 28844693 10.1016/j.cell.2017.07.027

[7] Morita H Kubo T Rückert B Ravindran A Soyka MB Rinaldi AO Sugita K Wawrzyniak M Wawrzyniak P Motomura K Tamari M Orimo K Okada N Arae K Saito K Altunbulakli C Castro-Giner F Tan G Neumann A Sudo K Induction of human regulatory innate lymphoid cells from group 2 innate lymphoid cells by retinoic acid. J Allergy Clin Immunol. 2019; 143: 2190–2201.e9. 30682454 10.1016/j.jaci.2018.12.1018

[8] Golebski K Layhadi JA Sahiner U Steveling-Klein EH Lenormand MM Li RCY Bal SM Heesters BA Vilà-Nadal G Hunewald O Montamat G He FQ Ollert M Fedina O Lao-Araya M Vijverberg SJH Maitland-van der Zee AH van Drunen CM Fokkens WJ Durham SR Shamji MH Induction of IL-10-producing type 2 innate lymphoid cells by allergen immunotherapy is associated with clinical response. Immunity. 2021; 54: 291–307.e7. 33450188 10.1016/j.immuni.2020.12.013

[9] Eljaszewicz A Ruchti F Radzikowska U Globinska A Boonpiyathad T Gschwend A Morita H Helbling A Arasi S Kahlert H Berek N Nandy A Akdis M Willers C Moniuszko M Akdis CA Sokolowska M Trained immunity and tolerance in innate lymphoid cells, monocytes, and dendritic cells during allergen-specific immunotherapy. J Allergy Clin Immunol. 2021; 147: 1865–1877. 33039478 10.1016/j.jaci.2020.08.042

[10] López-Abente J Benito-Villalvilla C Jaumont X Pfister P Tassinari P Palomares O Omalizumab restores the ability of human plasmacytoid dendritic cells to induce Foxp3+Tregs. Eur Respir J. 2021; 57: 2000751. 32675208 10.1183/13993003.00751-2020

[11] Soria I López-Relaño J Viñuela M Tudela JI Angelina A Benito-Villalvilla C Díez-Rivero CM Cases B Manzano AI Fernández-Caldas E Casanovas M Palomares O Subiza JL Oral myeloid cells uptake allergoids coupled to mannan driving Th1/Treg responses upon sublingual delivery in mice. Allergy. 2018; 73: 875–884. 29319882 10.1111/all.13396 PMC5947296

[12] Benito-Villalvilla C Perez-Diego M Subiza JL Palomares O Allergoid-mannan conjugates imprint tolerogenic features in human macrophages. Allergy. 2022; 77: 320–323. 34601743 10.1111/all.15118

[13] Sirvent S Soria I Cirauqui C Cases B Manzano AI Diez-Rivero CM Reche PA López-Relaño J Martínez-Naves E Cañada FJ Jiménez-Barbero J Subiza J Casanovas M Fernández-Caldas E Subiza JL Palomares O Novel vaccines targeting dendritic cells by coupling allergoids to nonoxidized mannan enhance allergen uptake and induce functional regulatory T cells through programmed death ligand 1. J Allergy Clin Immunol. 2016; 138: 558–567.e11. 27177779 10.1016/j.jaci.2016.02.029

[14] Benito-Villalvilla C Perez-Diego M Angelina A Kisand K Rebane A Subiza JL Palomares O Allergoid-mannan conjugates reprogram monocytes into tolerogenic dendritic cells via epigenetic and metabolic rewiring. J Allergy Clin Immunol. 2022; 149: 212–222.e9. 34153371 10.1016/j.jaci.2021.06.012

[15] Wen H Qu L Zhang Y Xu B Ling S Liu X Luo Y Huo D Li W Yao X A Dendritic Cells-Targeting Nano-Vaccine by Coupling Polylactic-Co-Glycolic Acid-Encapsulated Allergen with Mannan Induces Regulatory T Cells. Int Arch Allergy Immunol. 2021; 182: 777–787. 34289474 10.1159/000512872

[16] Angelina A Perez-Diego M Lopez-Abente J Ruckert B Nombela I Akdis M Martín-Fontecha M Akdis C Palomares O Cannabinoids induce functional Tregs by promoting tolerogenic DCs via autophagy and metabolic reprograming. Mucosal Immunol. 2022; 15: 96–108. 34548620 10.1038/s41385-021-00455-x PMC8732281

[17] Dolch A Kunz S Dorn B Alessandrini F Müller W Jack RS Martin SF Roers A Jakob T IL-10 signaling in dendritic cells is required for tolerance induction in a murine model of allergic airway inflammation. Eur J Immunol. 2019; 49: 302–312. 30566244 10.1002/eji.201847883

[18] Zemmour D Zilionis R Kiner E Klein AM Mathis D Benoist C Single-cell gene expression reveals a landscape of regulatory T cell phenotypes shaped by the TCR. Nat Immunol. 2018; 19: 291–301. 29434354 10.1038/s41590-018-0051-0 PMC6069633

[19] Scadding GW Shamji MH Jacobson MR Lee DI Wilson D Lima MT Pitkin L Pilette C Nouri-Aria K Durham SR Sublingual grass pollen immunotherapy is associated with increases in sublingual Foxp3-expressing cells and elevated allergen-specific immunoglobulin G4, immunoglobulin A and serum inhibitory activity for immunoglobulin E-facilitated allergen binding to B cells. Clin Exp Allergy. 2010; 40: 598–606. 20184605 10.1111/j.1365-2222.2010.03462.x

[20] Boonpiyathad T Sokolowska M Morita H Rückert B Kast JI Wawrzyniak M Sangasapaviliya A Pradubpongsa P Fuengthong R Thantiworasit P Sirivichayakul S Kwok WW Ruxrungtham K Akdis M Akdis CA Der p 1-specific regulatory T-cell response during house dust mite allergen immunotherapy. Allergy. 2019; 74: 976–985. 30485456 10.1111/all.13684

[21] Satitsuksanoa P Daanje M Akdis M Boyd SD van de Veen W Biology and dynamics of B cells in the context of IgE-mediated food allergy. Allergy. 2021; 76: 1707–1717. 33274454 10.1111/all.14684

[22] Jansen K Cevhertas L Ma S Satitsuksanoa P Akdis M van de Veen W Regulatory B cells, A to Z. Allergy. 2021; 76: 2699–2715. 33544905 10.1111/all.14763

[23] Ma S Satitsuksanoa P Jansen K Cevhertas L van de Veen W Akdis M B regulatory cells in allergy. Immunol Rev. 2021; 299: 10–30. 33345311 10.1111/imr.12937

[24] van de Veen W Stanic B Yaman G Wawrzyniak M Söllner S Akdis DG Rückert B Akdis CA Akdis M IgG4 production is confined to human IL-10-producing regulatory B cells that suppress antigen-specific immune responses. J Allergy Clin Immunol. 2013; 131: 1204–1212. 23453135 10.1016/j.jaci.2013.01.014

[25] Boonpiyathad T Meyer N Moniuszko M Sokolowska M Eljaszewicz A Wirz OF Tomasiak-Lozowska MM Bodzenta-Lukaszyk A Ruxrungtham K van de Veen W High-dose bee venom exposure induces similar tolerogenic B-cell responses in allergic patients and healthy beekeepers. Allergy. 2017; 72: 407–415. 27341567 10.1111/all.12966

[26] Boonpiyathad T van de Veen W Wirz O Sokolowska M Rückert B Tan G Sangasapaviliya A Pradubpongsa P Fuengthong R Thantiworasit P Sirivichayakul S Ruxrungtham K Akdis CA Akdis M Role of Der p 1-specific B cells in immune tolerance during 2 years of house dust mite-specific immunotherapy. J Allergy Clin Immunol. 2019; 143: 1077–1086.e10. 30529452 10.1016/j.jaci.2018.10.061

[27] Bosma A Abdel-Gadir A Isenberg DA Jury EC Mauri C Lipid-antigen presentation by CD1d(+) B cells is essential for the maintenance of invariant natural killer T cells. Immunity. 2012; 36: 477–490. 22406267 10.1016/j.immuni.2012.02.008 PMC3391684

[28] Matsuoka T Shamji MH Durham SR Allergen immunotherapy and tolerance. Allergol Int. 2013; 62: 403–413. 24280670 10.2332/allergolint.13-RAI-0650

[29] Shamji MH Valenta R Jardetzky T Verhasselt V Durham SR Würtzen PA van Neerven RJJ The role of allergen-specific IgE, IgG and IgA in allergic disease. Allergy. 2021; 76: 3627–3641. 33999439 10.1111/all.14908 PMC8601105

[30] Shamji MH Larson D Eifan A Scadding GW Qin T Lawson K Sever ML Macfarlane E Layhadi JA Würtzen PA Parkin RV Sanda S Harris KM Nepom GT Togias A Durham SR Differential induction of allergen-specific IgA responses following timothy grass subcutaneous and sublingual immunotherapy. J Allergy Clin Immunol. 2021; 148: 1061–1071.e11. 33819508 10.1016/j.jaci.2021.03.030 PMC12400438

[31] van de Veen W Akdis M Role of IgG4 in IgE-mediated allergic responses. J Allergy Clin Immunol. 2016; 138: 1434–1435. 27566454 10.1016/j.jaci.2016.07.022

[32] Heeringa JJ McKenzie CI Varese N Hew M Bakx ATCM Aui PM Rolland JM O’Hehir RE van Zelm MC Induction of IgG2 and IgG4 B-cell memory following sublingual immunotherapy for ryegrass pollen allergy. Allergy. 2020; 75: 1121–1132. 31587307 10.1111/all.14073 PMC7317934

[33] Shamji MH Kappen J Abubakar-Waziri H Zhang J Steveling E Watchman S Kouser L Eifan A Switzer A Varricchi G Marone G Couto-Francisco NC Calderon M Durham SR Nasal allergen-neutralizing IgG4 antibodies block IgE-mediated responses: Novel biomarker of subcutaneous grass pollen immunotherapy. J Allergy Clin Immunol. 2019; 143: 1067–1076. 30445057 10.1016/j.jaci.2018.09.039

[34] Boonpiyathad T Pradubpongsa P Mitthamsiri W Satitsuksanoa P Jacquet A Sangasapaviliya A Allergen-specific immunotherapy boosts allergen-specific IgD production in house dust mite-sensitized asthmatic patients. Allergy. 2020; 75: 1457–1460. 31769883 10.1111/all.14133

[35] Shan M Carrillo J Yeste A Gutzeit C Segura-Garzón D Walland AC Pybus M Grasset EK Yeiser JR Matthews DB van de Veen W Comerma L He B Boonpiyathad T Lee H Blanco J Osborne LC Siracusa MC Akdis M Artis D Secreted IgD Amplifies Humoral T Helper 2 Cell Responses by Binding Basophils via Galectin-9 and CD44. Immunity. 2018; 49: 709–724.e8. 30291028 10.1016/j.immuni.2018.08.013 PMC6366614

[36] Jensen-Jarolim E Roth-Walter F Jordakieva G Pali-Schöll I Allergens and Adjuvants in Allergen Immunotherapy for Immune Activation, Tolerance, and Resilience. J Allergy Clin Immunol Pract. 2021; 9: 1780–1789. 33753052 10.1016/j.jaip.2020.12.008

[37] Benito-Villalvilla C Soria I Subiza JL Palomares O Novel vaccines targeting dendritic cells by coupling allergoids to mannan. Allergo J Int. 2018; 27: 256–262. 30546997 10.1007/s40629-018-0069-8 PMC6267119

[38] Benito-Villalvilla C Soria I Pérez-Diego M Fernández-Caldas E Subiza JL Palomares O Alum impairs tolerogenic properties induced by allergoid-mannan conjugates inhibiting mTOR and metabolic reprogramming in human DCs. Allergy. 2020; 75: 648–659. 31494959 10.1111/all.14036 PMC7079174

[39] Subiza JL Palomares O Quinti I Sánchez-Ramón S Editorial: Trained Immunity-Based Vaccines. Front Immunol. 2021; 12: 716296. 34249020 10.3389/fimmu.2021.716296 PMC8264451

[40] Wanka L Jappe U Trained immunity and allergy: State of the art and future perspectives. Allergy. 2021; 76: 1265–1267. 33037616 10.1111/all.14617

